# Endothelialization of Whey Protein Isolate-Based Scaffolds for Tissue Regeneration

**DOI:** 10.3390/molecules28207052

**Published:** 2023-10-12

**Authors:** Hatice Genç, Bernhard Friedrich, Christoph Alexiou, Krzysztof Pietryga, Iwona Cicha, Timothy E. L. Douglas

**Affiliations:** 1 Section of Experimental Oncology und Nanomedicine (SEON), Else Kröner-Fresenius-Stiftung-Professorship, Department of Otorhinolaryngology, Head and Neck Surgery, Universitätsklinikum Erlangen, University of Erlangen-Nürnberg, 91054 Erlangen, Germany; hatice.genc@uk-erlangen.de (H.G.);; 2 Silesian Park of Medical Technology Kardio-Med Silesia, 41-800 Zabrze, Poland; volger333@gmail.com; 3 School of Engineering, Lancaster University, Lancaster LA1 4YX, UK

**Keywords:** whey protein, endothelial cell compatibility, thiol levels, hydrogels, tubular scaffolds, 3D cell seeding

## Abstract

Background: Whey protein isolate (WPI) is a by-product from the dairy industry, whose main component is β-lactoglobulin. Upon heating, WPI forms a hydrogel which can both support controlled drug delivery and enhance the proliferation and osteogenic differentiation of bone-forming cells. This study makes a novel contribution by evaluating the ability of WPI hydrogels to support the growth of endothelial cells, which are essential for vascularization, which in turn is a pre-requisite for bone regeneration. Methods: In this study, the proliferation and antioxidant levels in human umbilical vascular endothelial cells (HUVECs) cultured with WPI supplementation were evaluated using real-time cell analysis and flow cytometry. Further, the attachment and growth of HUVECs seeded on WPI-based hydrogels with different concentrations of WPI (15%, 20%, 30%, 40%) were investigated. Results: Supplementation with WPI did not affect the viability or proliferation of HUVECs monitored with real-time cell analysis. At the highest used concentration of WPI (500 µg/mL), a slight induction of ROS production in HUVECs was detected as compared with control samples, but it was not accompanied by alterations in cellular thiol levels. Regarding WPI-based hydrogels, HUVEC adhered and spread on all samples, showing good metabolic activity. Notably, cell number was highest on samples containing 20% and 30% WPI. Conclusions: The demonstration of the good compatibility of WPI hydrogels with endothelial cells in these experiments is an important step towards promoting the vascularization of hydrogels upon implantation in vivo, which is expected to improve implant outcomes in the future.

## 1. Introduction

Hydrogels, or 3D cross-linked polymer networks containing entrapped water, are interesting materials for applications in tissue engineering due to their high water content and elasticity, allowing deformation to mimic the changes in shape of native tissue [1]. Recent years brought about an increasing interest in biomaterials based on natural proteins and polymers, both of animal and plant origin [2,3], such as gelatin, fibrin [4], keratin [5] or silk fibroin and marine polysaccharides [6], but also waste raw materials, such as, e.g., lignin [7,8] or casein [9], which are environmentally friendly, inexpensive and widely available [10]. Whey protein isolate (WPI), a by-product from the dairy industry [11], was proposed as a biomaterial suitable for bone tissue regeneration [12], wound healing [13,14] and drug delivery [15,16,17,18]. WPI, whose main component is β-lactoglobulin (β-LG) (75% by dry mass), has the unique ability to form a three-dimensional hydrogel network without the use of potentially toxic cross-linking agents. This process is based solely on the heating of an aqueous solution of WPI above 60 °C, which leads to unfolding of the β-LG and the formation of interprotein bonds [19,20]. Moreover, its cytocompatibility [21], anti-inflammatory and antioxidant [21,22] properties all make WPI a promising material for use in biomedical applications.

Previous studies indicated a beneficial effect of enzymatically hydrolyzed whey on the antioxidant capacities of endothelial cells, a cell type known for its sensitivity to biomaterial-induced oxidative stress [23]. Significantly increased intracellular glutathione (GSH) levels and catalase activity were observed in human umbilical vein endothelial cells (HUVECs) incubated with hydrolyzed whey compared with cells cultured in media alone [24]. In endothelial cells exposed to diabetic conditions, treatment with whey hydrolysate prevented mitochondrial injury and redox imbalance [25]. Increased GSH levels in plasma and erythrocytes have also been reported in rats which were fed a diet supplemented with 10% whey proteins [26,27], but the evidence for its protective effects on vascular functions in humans remains unsubstantiated [28] Regarding tissue engineering applications, several reports have indicated that WPI dissolved in cell culture medium stimulates the osteogenic differentiation of bone-forming cells [12,29,30,31]. However, in the majority of the previous studies, WPI has been used only as a supplement for cell culture medium, in the form of lysate or dissolved powder. Thus far, interactions of primary human cells with WPI-based hydrogels have mainly focused on bone-forming cells [21,32]. Vascularization is known to be a prerequisite for bone tissue growth; hence, it is important to determine if WPI hydrogels promote angiogenesis upon implantation.

In this study, we used WPI as a cell medium supplement in the form of a hydrogel to address its compatibility with primary human endothelial cells. Initially, we investigated the effect of WPI supplementation on viability and antioxidant levels in HUVECs. Subsequently, we evaluated for the first time the capacity of WPI-based scaffolds to support the growth of endothelial cells seeded on hydrogel surfaces.

## 2. Results

### 2.1. Effect of WPI Supplementation on Proliferation and Antioxidant Levels in HUVECs

**Figure 1:**
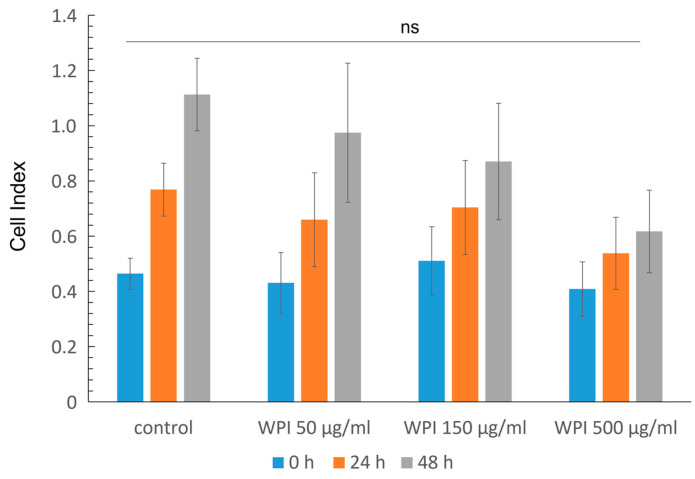
Effects of WPI supplementation on primary HUVECs. Graph shows the results of impedance-based real-time monitoring of Cell Index, reflecting cell number, attachment and viability. HUVECs were treated with the indicated concentrations of WPI and monitored for 48 h after WPI administration (*n* = 3 independent experiments, each with hexaplicate samples). Control, medium only; ns, not significant.

In the first part of the study, we investigated whether the supplementation of cell culture medium with WPI affects the viability of primary HUVECs. Real-time cell analysis based on impedance measurements was used to determine the so-called Cell Index, which reflects the cell number, attachment and viability. As shown in Figure 1, the supplementation of cell culture media with 50, 150 or 500 µg/mL of WPI had no significant effect on the Cell Index, which indicated that the viability, proliferation and attachment of HUVECs were unchanged in the presence of WPI.

**Figure 2:**
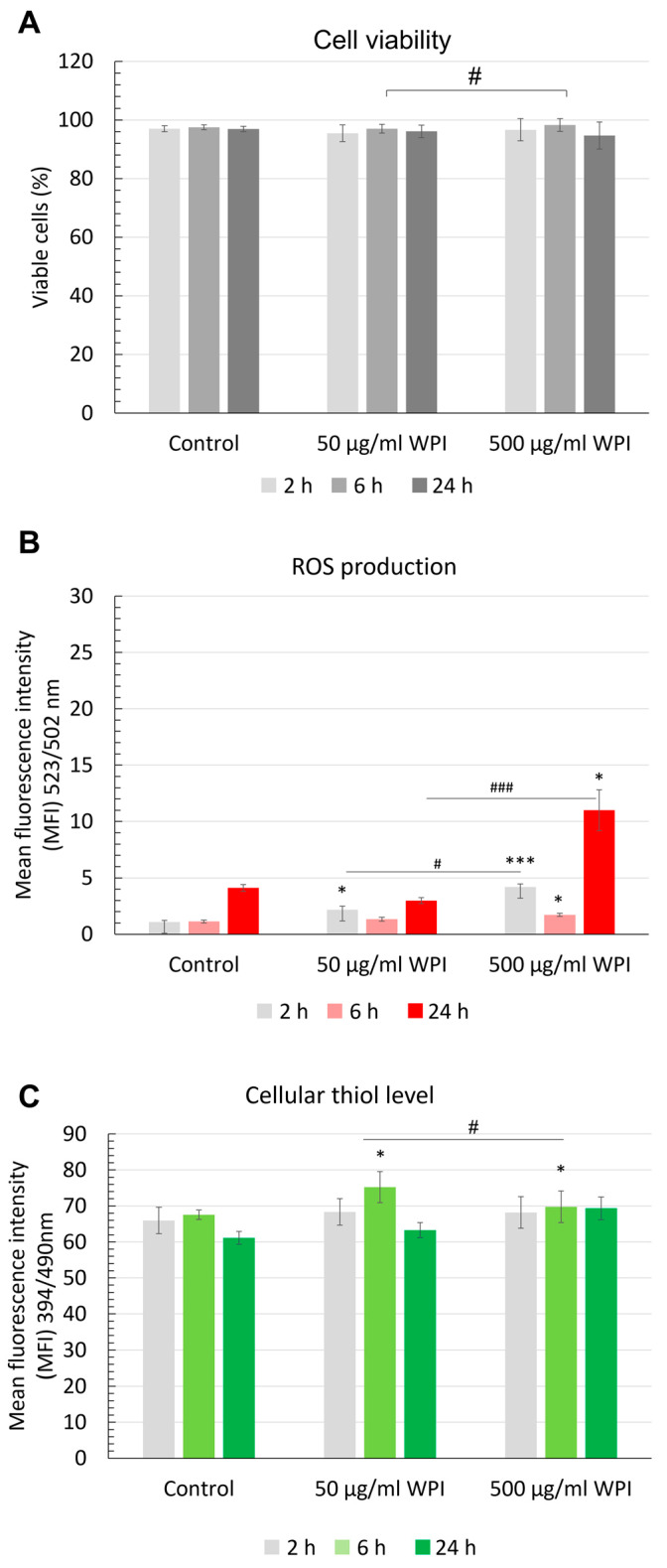
Flow cytometric analysis of (**A**) HUVEC viability, (**B**) intracellular reactive oxygen species (ROS) levels and (**C**) cellular thiol levels. HUVECs seeded in 24-well plates were supplemented with WPI at indicated concentrations. The flow cytometric analyses were performed after 2 h, 6 h or 24 h of incubation. Graphs show data from *n* = 3 independent experiments, each with triplicate samples. * *p* < 0.05, *** *p* < 0.001 versus control (without WPI); ^#^ *p* < 0.05, ^###^ *p* < 0.001: 50µg/mL WPI versus 500 µg/mL WPI at respective time points.

In the further experiments, we therefore selected two WPI concentrations (50 and 500 µg/mL) to investigate their effect on intracellular reactive oxygen species (ROS) production and antioxidant levels. Primary HUVECs are very sensitive to stimuli and respond to oxidative stress-inducing biomaterials, with rapid antioxidant depletion within 6 h and subsequent necrotic cell death [23]. The flow cytometric analyses confirmed that cell viability was not affected by WPI supplementation independently of concentration and remained constantly above 90% (Figure 2A). In all samples, the levels of cellular ROS were very low and only a slight increase in ROS production was detected after 24 h of cell culture, independently of the WPI concentration (Figure 2B). At the highest used concentration of WPI (500 µg/mL), the induction of ROS production in HUVECs was nearly three-fold higher as compared with control samples (*p* < 0.05), but the signal intensity values remained low (mean 4.1 in control vs. 11 in samples treated with 500 µ/mL WPI). There was no major effect of WPI supplementation on cellular thiols. A very minor and transient increase in antioxidant level was observed after 6 h in cells supplemented with 50 µg/mL WPI, followed by a decrease to control levels after 24 h, which paralleled the induction of ROS at that time point. Thiol levels of HUVECs treated with 500 µg/mL WPI remained nearly constant during 24 h of culture (Figure 2C).

Following the initial evaluation of the effects of cell culture media supplementation with WPI on primary HUVECs, we subsequently aimed to determine the cell–material interactions between HUVECs and WPI-based scaffolds in the form of hydrogels.

### 2.2. Endothelialization of Flat WPI Hydrogels

**Figure 3:**
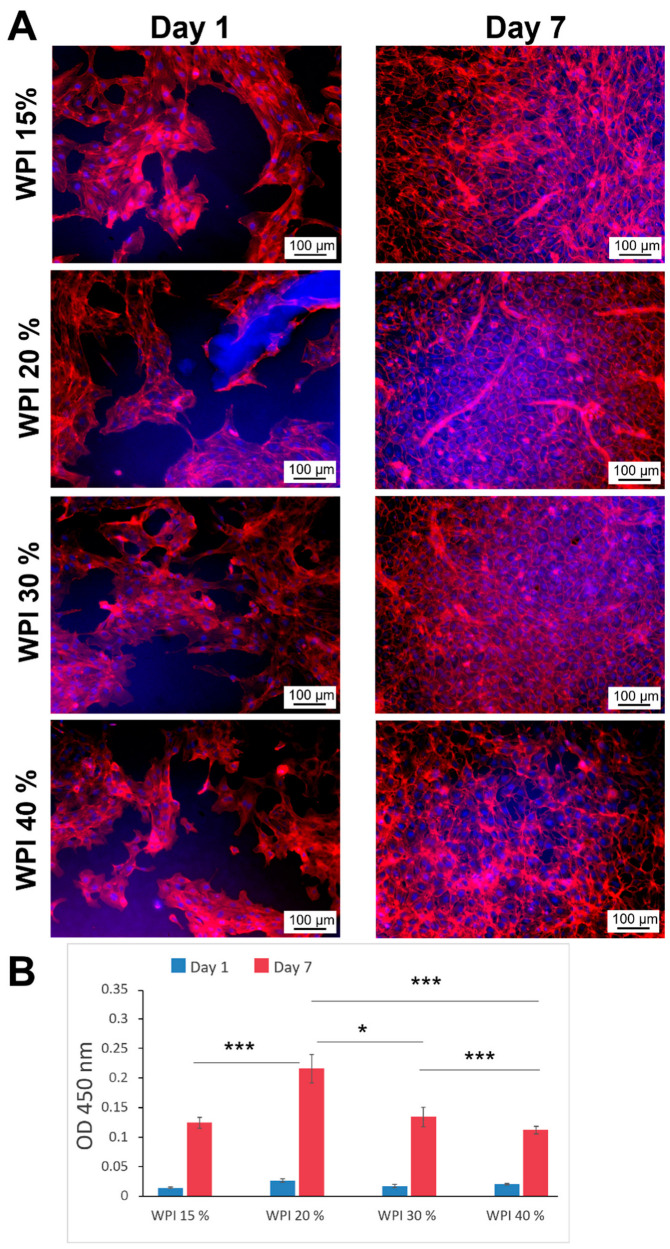
Endothelial cell morphology (**A**) and metabolic activity (**B**) after 1 or 7 days of culture on flat WPI scaffolds. To visualize cell morphology, HUVECs grown on WPI hydrogels were stained with rhodamine phalloidin (F-actin fibers) and Hoechst (nuclei). Metabolic activity was measured prior to cell fixation using WST-8 assay, based on the extracellular reduction of WST-8 by NADH produced in the mitochondria and resulting in an orange-colored formazan which dissolved directly into the culture medium. Graph shows mean ± SD of *n* = 3 independent experiments, *** *p* < 0.001, * *p* < 0.05.

**Figure A1:**
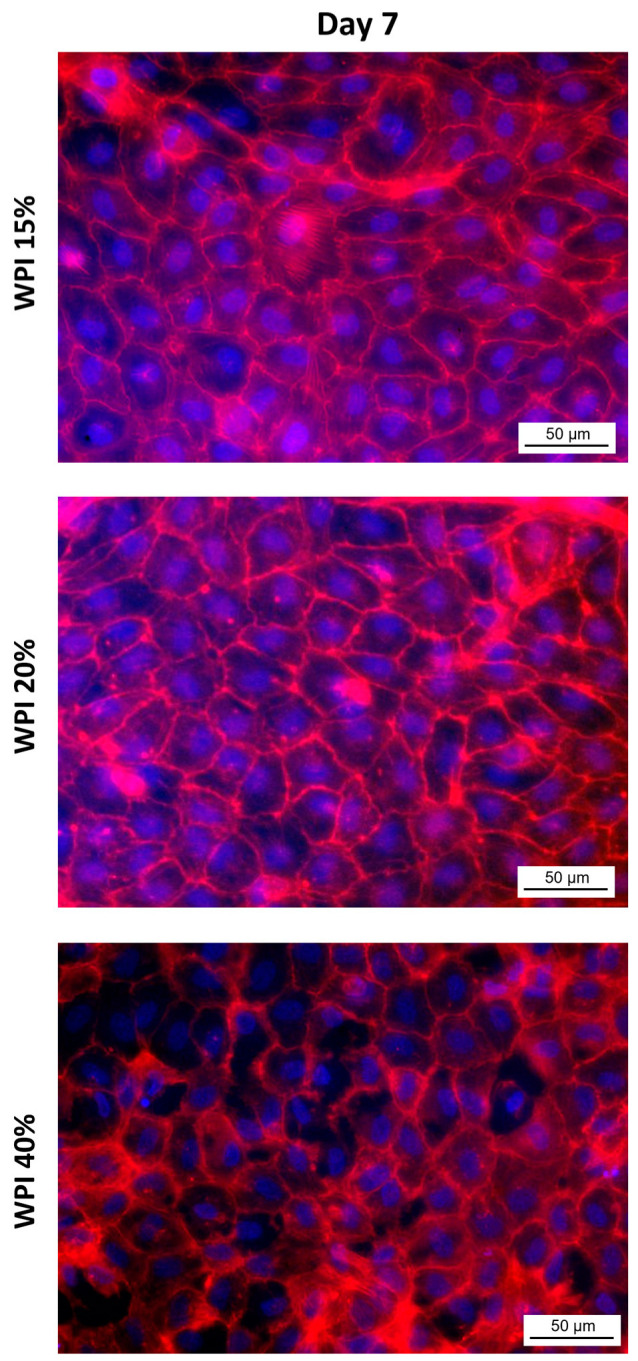
Morphology of primary HUVECs grown on WPI hydrogels for 7 days.

The capacity of WPI-based scaffolds to support endothelial cell growth has not been investigated thus far. To address this question, hydrogels containing different WPI concentrations (15, 20, 30 and 40%) were prepared as described in the Materials and Methods section (Section 4.5) and seeded with primary endothelial cells. As shown in Figure 3, the attachment of HUVECs to all substrates was similar, independent of the used WPI concentration (in the range of 15–40%). Importantly, the cells started to spread and form monolayers already on Day 1. On Day 7, the best growth and spreading was observed on WPI-20% and WPI-30% scaffolds, which showed a dense, confluent monolayer of endothelial cells with a native-like, cobblestone morphology (Figure 3). In comparison, the cells grown on the WPI-40% scaffold were marginally less confluent on Day 7, and slight changes in their morphology became evident (see Appendix A, Figure A1). The evaluation of mitochondrial activity using the WST-8 assay demonstrated an elevated NADH production in the mitochondria, reflecting a strong increase in cell metabolism over time and corresponding to cell proliferation on the WPI substrates. We observed a significantly higher metabolic activity of HUVECs on WPI-20% compared with other hydrogels. Because of this, the hydrogel containing 20% WPI was selected as a substrate for further characterization, preparing 3D scaffolds and performing studies on cell seeding in tubular constructs.

### 2.3. WPI Hydrogel Characterization

Compressive testing of 20% WPI hydrogels revealed the following mechanical properties, all expressed as mean ± standard deviation. Young’s modulus of the hydrogels was 0.17 ± 0.02 MPa, compressive strength was 0.68 ± 0.32 MPa and compressive strain at break was 67.0 ± 0.5%.

**Figure 4:**
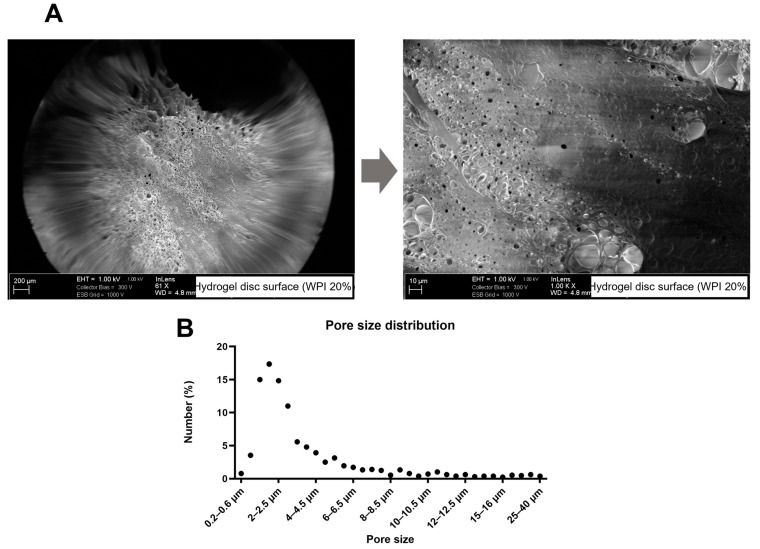
SEM images of freeze-dried WPI-20% hydrogel discs (**A**) and distribution of pore sizes in hydrogel (**B**). Images taken at different magnifications show hydrogel surface structure and porosity. The analysis of pore sizes (number of counts = 1392) shows that above 60% of pores were smaller than 3 µm in size.

The analysis of freeze-dried WPI-20% hydrogel samples with scanning electron microscopy indicated that the surface of the hydrogels was porous and presented considerable microscale roughness in the dry state (Figure 4A). It should be mentioned that the morphology in the dry state is not necessarily representative of the morphology in the wet state. The SEM-image analysis showed that the pore dimensions were between 0.26 and 35.43 µm (mean pore size 2.6 µm, median 1.6 µm, counts = 1392) and had a porosity of 3.71%. The pore size distribution is shown in Figure 4B.

### 2.4. Endothelialization of Tubular WPI Constructs

**Figure 5:**
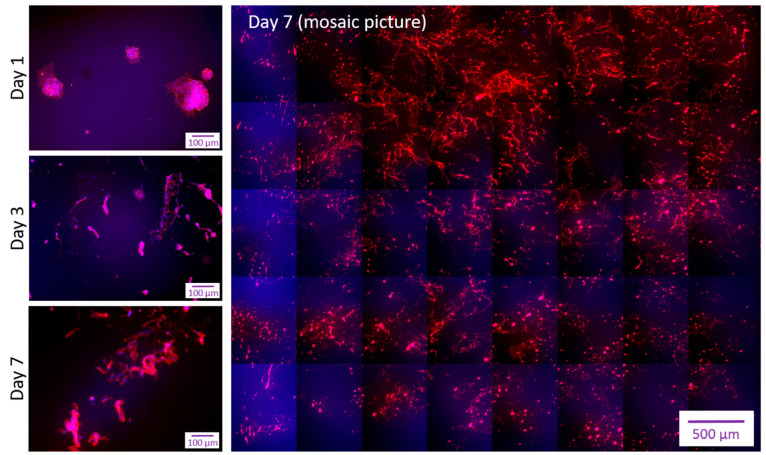
Endothelial cell morphology 1, 3 or 7 days after seeding on tubular WPI-20% scaffolds using a rolling mixer. The produced WPI tubes were filled with primary HUVECs and placed on a roller mixer for 40 min to allow cell attachment. Afterwards, scaffolds were incubated for up to 7 days to observe the endothelial cell morphology. The lower magnification image (×10) shows the whole luminal surface of the scaffold. Cells were stained with rhodamine phalloidin (F-actin fibers) and Hoechst (nuclei). Representative images of *n* = 3 independent experiments are shown.

To test whether the WPI-based scaffolds support endothelialization equally well in 3D geometries, tubular scaffolds containing WPI-20% (see 4.11) were prepared. In the first set of experiments, the produced tubes were filled with primary (unlabeled) HUVECs and placed on a roller mixer for 40 min to allow for cell attachment. As shown in Figure 5, the cell morphology on Day 1 was different from that of the flat samples (see Figure 4A above). The cells showed non-uniform distribution with clustered islets and distinct areas of more spread morphology. Slightly improved monolayer formation and cell morphology were observed on Day 3, but by Day 7, a noticeable monolayer detachment accompanied by a spindle-like cell morphology occurred (Figure 5).

**Figure 6:**
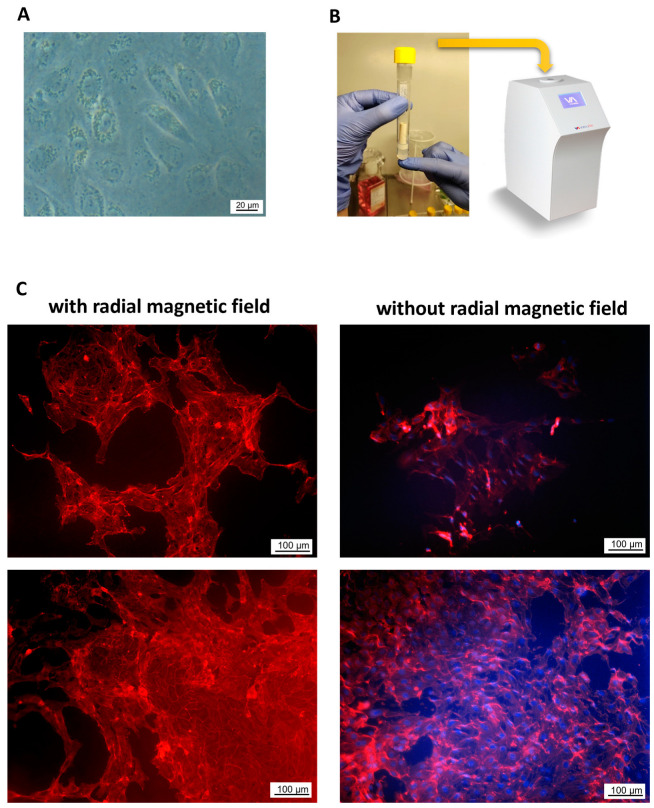
Magnetic cell seeding on tubular WPI-20% scaffolds. HUVECs were pre-incubated with SPIONs to render them magnetically responsive and were seeded on the lumens of the vertically positioned scaffolds using a radial magnetic force. (**A**) Light microscopy image of endothelial cells loaded with SPION^LA^; (**B**) magnetic cell seeding set-up; (**C**) example cell-covered areas after 7 days of culture. Left panel: HUVECs seeded using radial magnetic field; right panel: HUVECs seeded without radial magnetic field. Representative images of *n* = 3 independent experiments are shown.

To test whether the endothelialization of tubular scaffolds can be improved using the magnetic cell seeding technique, which was previously shown to improve seeding density and uniformity, a separate set of experiments was performed with magnetically labeled HUVECs. For this purpose, HUVECs were pre-incubated with superparamagnetic iron oxide nanoparticles (SPIONs) to render them magnetically responsive, and cells were subsequently seeded on the lumens of the vertically positioned scaffolds using a radial magnetic force for 15 min (Figure 6A,B). The efficacy of endothelialization and cell morphology were evaluated on the 3D scaffolds after 7 days. As shown in Figure 6C, rather small regions of monolayer-forming cells were detectable on the 3D scaffolds 7 days after magnetic cell seeding. This is in contrast to the cell coverage observed in the 2D conditions (shown in Figure 3A). Although no major monolayer detachment was observed, the cell growth was slow and there were visible amounts of internalized particles still present in the cells, indicative of their low metabolic activity.

## 3. Discussion

WPI is a natural, protein-rich material which has been proposed as a building block for scaffolds to support bone regeneration [12,21]**,** among other potential tissue-engineering applications [10,33]. Its interactions with bone cells and stem cells have been previously investigated, indicating good biocompatibility, as well as antimicrobial and antioxidant properties. As human endothelial cells are essential contributors to graft vascularization and survival, we aimed to investigate the effects of cell culture media supplementation with WPI, as well as the cell–matrix interactions of primary HUVECs and WPI. Expectedly, the supplementation of cell culture media with concentrations up to 500 µg/mL WPI had no significant effect on the cell viability, proliferation or attachment of HUVECs in real-time cell analysis. Flow cytometry confirmed that cell viability was not affected by WPI supplementation. WPI was previously reported to induce antioxidant synthesis in HUVECs [22,24] and improve metabolic redox status in endothelial cells exposed to diabetic conditions [25]. We therefore analyzed the cellular production of ROS upon WPI supplementation and compared it with the level of thiols over 24 h of culture. In the presence of oxidative stress-inducing materials, cellular antioxidants in primary endothelial cells are rapidly depleted, which leads to necrotic cell death within 6 h [23]. Our studies showed that the levels of cellular ROS were very low after 2 h and 6 h of culture, both in controls and WPI-supplemented samples. After 24 h of culture, a minor increase in ROS production was detected in all samples. Although the median ROS production in HUVECs supplemented with 500 µg/mL WPI (median MFI 7,56; range 6.60–17.81) was nearly twice as high as compared with control samples (median MFI 4.43; range 3.39–5.15), the ROS signal intensity values remained in the overall low range. Relatively large standard error values further point to a possible inter-donor variability in the HUVEC response to increased concentrations of the supplement. Only a transient increase in antioxidant level was observed after 6 h in cells supplemented with 50 µg/mL WPI, followed by a decrease to control levels after 24 h, which paralleled the slight induction of ROS at that time point. Previously reported studies [24] indicated that WPI supplementation may increase the levels of antioxidants in HUVECs. However, in those reports, cells were cultured for extended periods of time, while we monitored the short-term effects of WPI supplementation. It must also be noted that cell responses may differ in the presence of oxidative stimulus, which was not investigated in the present study.

In contrast to WPI supplementation [28,34], little is known about the interactions of endothelial cells with WPI-based hydrogels, as previous studies focused mainly on bone-forming cells or stem cells. Our results demonstrated that the flat hydrogel samples supported the attachment, spreading and proliferation of primary HUVECs. The cells gained a native-like cobblestone morphology and started to form a monolayer already on Day 1 post-seeding. After 7 days of culture, the best morphological features and metabolic activity were observed on WPI-20% scaffolds. Although HUVECs seeded on stiffer substrates (WPI-40%) remained viable and attached to the hydrogels, their morphology was more irregular and characterized by pronounced F-actin stress fibers, and the surface coverage was less uniform, in line with the reported observations of stiffening matrices [35]. Notably, the average value of the Young’s modulus of the WPI-20% hydrogels (170 kPa) was approximately one-fifth or less that measured for WPI-40% hydrogels in previous studies [21,32]. In another report, 30% WPI hydrogels containing 8% curdlan also showed a Young’s modulus value approximately five times higher than the value measured for WPI-20% hydrogels in the present study [36]. In those studies, the WPI hydrogels were intended as scaffolds for bone or osteochondral tissue regeneration, and it was previously shown that stiff hydrogels support the attachment of bone-forming or cartilage-forming cells [37,38,39]. Generally, angiogenic processes and the growth of vascular cells may be inhibited in such stiff scaffolds [35,40], which could pose a problem for regenerative approaches that require tissue vascularization to supply engineered constructs with oxygen and nutrients. In our study, in spite of the slightly slower growth of HUVECs on WPI-40% hydrogels, a good compatibility of the material with human endothelium was confirmed, which may suggest that with progressive material degradation and increasing porosity, the WPI-40% scaffolds can potentially be vascularized. Potentially, scaffolds with a WPI concentration gradient, or those combining hydrogels of different stiffness ranges, could also enable fast colonization with different cell populations. In comparison with scaffolds for bone regeneration, soft tissue regeneration, such as vascular tissue engineering, requires less-stiff materials [37,41]. Our data indicate that by reducing the WPI content, the Young’s modulus of WPI hydrogels can be tailored to a value more suitable for, e.g., vascular tissue engineering. The determination of the porosity, pore size distribution and surface roughness of a hydrogel biomaterial in the wet state is not trivial, and the development of advanced techniques to evaluate these properties would be useful in understanding cell–hydrogel interactions. However, such studies would be worthy of a paper in their own right and are outside the scope of this study.

**Figure 7:**
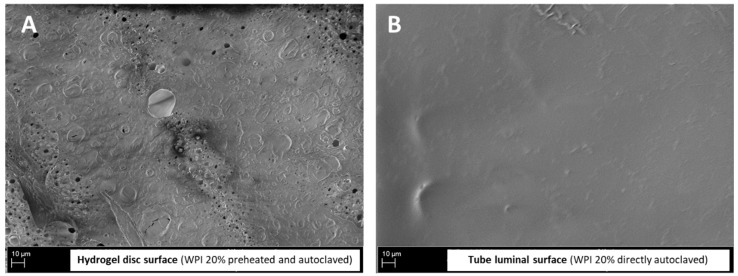
SEM images showing the surfaces of (**A**) hydrogel disc and (**B**) tube lumen of WPI-20% scaffolds.

Because the best HUVEC growth was observed on scaffolds containing 20% WPI, we additionally attempted to produce 3D tubular scaffolds in order to investigate their potential as a material for vascular grafts. However, we did not achieve efficient endothelialization of 3D constructs, neither using the rotational method nor with the magnetic cell seeding approach. Compared with endothelial cell growth on 2D surfaces, cell attachment to tubular constructs was clearly weaker, and only irregularly distributed areas of monolayer were observed. This was likely due to the differences in hydrogel surface properties between flat hydrogels and 3D samples resulting from the fabrication method. The 3D scaffolds were produced using the tube-in-the-tube method and had, therefore, a very smooth luminal surface, as shown in Figure 7. This type of surface may prevent stable cell attachment even during magnetic seeding, or—as observed in the case of rotational seeding—result in cell detachment after 7 days [42,43]. Therefore, if this potentially promising material should be used in the future for vascular tissue engineering, it would be necessary to develop an improved production method for 3D scaffolds to provide the cells with a suitable surface nano-/microstructure [44].

## 4. Materials and Methods

### 4.1. Whey Protein Isolate Supplementation

WPI (BiPro, 97.7% of protein and 75% of β-LG by dry mass) was obtained from Davisco Foods International Inc., Le Sueur, MN, USA). For cell culture studies, WPI powder was weighed and dissolved in endothelial cell growth medium (Promo Cell, Heidelberg, Germany). After sterile filtering to remove residue, the stock solution (1 g/L) was diluted with medium to obtain appropriate end-concentrations.

### 4.2. Cell Isolation and Culture

Human umbilical vein endothelial cells (HUVECs) were isolated from freshly collected umbilical cords using a standard technique. Isolated cells were cultured in endothelial cell growth medium with endothelial cell growth supplement (Promo Cell, Heidelberg, Germany) containing 5% fetal calf serum, 4 µL/mL heparin, 10 ng/mL epidermal growth factor and 1 µg/mL hydrocortisone in a humidified 5% CO_2_ incubator. The use of human material was approved by the Institutional Ethical Committee on Human Research at the University Hospital Erlangen (ethical review number 85_14 B, from 21.02.2017). All subjects enrolled in this research gave Informed Consent according to the ethical guidelines. In all experiments, HUVECs at passages 1–2 were used.

### 4.3. Real-Time Cell Analysis

For monitoring the effects of nanoparticles on HUVEC viability, a xCELLigence system (RTCA DP Analyzer, Roche Diagnostics, Mannheim, Germany) was used. Experiments were performed in 16-well E-plates containing microelectrodes for impedance measurement (ACEA Bioscience, San Diego, CA, USA).

For the background measurement, 100 µL of cell-free endothelial cell growth medium was added to the wells. Afterwards, 50 µL of media from each well were replaced with 50 µL of cell suspension containing 1 × 10^3^ HUVECs and monitoring of impedance was initiated. At 24 h after seeding, an additional 100 µL of media containing WPI at concentrations 2x higher than the required final concentrations was added. The final WPI concentrations were as follows: 0, 50, 150 and 500 µg/mL. Cell growth was monitored every 10 min for 96 h. The experiments were performed in hexaplicate.

### 4.4. Flow Cytometric Analyses

Flow cytometry was performed using a Gallios cytofluorometer™ (Beckman Coulter, Fullerton, CA, USA) in order to analyze cell viability, apoptotic and necrotic cell numbers, intracellular ROS generation and cellular thiol content of HUVECs treated with WPI-supplemented medium.

#### 4.4.1. Preparation of 2′,7′-Dichlorofluorescin Diacetate (DCFH-DA) Probe for Intracellular ROS Detection

Detection of intracellular ROS was performed using 20,70-Dichlorofluorescin diacetate (DCFH-DA; Merck (Sigma-Aldrich), Darmstadt, Germany), which is de-esterified intracellularly upon oxidation and turns to highly fluorescent 2′,7′-dichlorofluorescein (DCF). This allows for a sensitive and rapid quantification of ROS in response to oxidative metabolism of cells and can be detected at an emission wavelength of 523 nm and an excitation of 502 nm. Prior to adding WPI, HUVECs were stained with DCFH-DA dye at 20 µM final concentration via 20 min incubation in a dark chamber at 5% CO_2_ and 37 °C. After incubation, cells were washed with PBS (10 mL) and seeded into 24-well plates. The medium was supplemented with 50, 150 or 500 µg/mL sterile WPI. WPI-untreated cells with and without a DCFH-DA label were used as controls. After the desired incubation period (6 h, 24 h, 72 h), cells were harvested and transferred to 15 mL falcon tubes and centrifuged at 300× *g* for 10 min. Cell pellets were re-suspended in 250 µL PBS, counted using MUSE^®^ Cell Analyzer and transferred into flow cytometer tubes (5 × 10^4^ cells/per sample).

#### 4.4.2. Preparation of Staining Solutions and Analysis of Flow Cytometry Data

To prepare the staining solution, 5.1 µg/mL DiI (1,1′,3,3,3′,3′-hexamethylindodicarbo-cyanine iodide [DiIC1(5)]), Life Technologies (Darmstadt, Germany), 20 µg/mL PI (propidium iodide, Merck) and 50 µM MBB (monobromobimane, 3-(bro momethyl)-2,5,6-trimethyl-1H,7H-pyrazolo [1,2-a]pyrazole-1,7-dione), Thermo Fisher Scientific, Schwerte, Germany) were dissolved in Ringer’s solution. Cell suspensions containing 5 × 10^4^ cells in 50 µL volume were mixed with 250 µL freshly prepared staining solution and incubated for 30 min at 37 °C. DiI dye stains the cells that only have intact mitochondrial membrane potential, indicating cell viability, while PI dye labels necrotic cells. Apoptotic cells are determined via gating on the area that is not stained by either DiI or PI dye in the whole cell population. Thus, viable cells were characterized by DiI-positive and PI-negative staining, apoptotic cells were DiI-negative and PI-negative and necrotic cells were DiI-negative and PI-positive. MBB dye gives a fluorescent signal upon coupling with thiols, including glutathione (GSH), N-acetylcysteine, mercaptopurine, peptides and plasma thiols, and the resulting thiol conjugate of monobromobimane has absorption/emission maxima ~394/490 nm. The side scatter value of control cells was set to 100%, and effects of the tested hydrogels were calculated with reference to that. Mean values of DCF (oxidized form of DCFH-DA) and MBB were calculated using the area that was gated at viable cells. Every sample was measured for a fixed time (40 s, per sample). Electronic compensation was used to correct for bleed-through emission. The data analysis was performed with Kaluza software version 2.0 (Beckman Coulter). All flow cytometry analyses were conducted in three independent experiments, each with triplicate samples. Control cells with DCFH-DA were represented as control data in all graphs.

### 4.5. WPI Hydrogel Preparation

WPI hydrogels were made as described previously [32]. Briefly, 15, 20, 30 and 40 wt/vol% aqueous WPI solutions were prepared by mixing the appropriate mass of WPI powder vigorously with Milli-Q water in a 50 mL Falcon tube. Mixtures were placed in the fridge overnight at 4 °C to allow foam to settle. Hydrogels were formed by transferring them to 2 mL Eppendorf tubes and heating at 100 °C for 5 min.

### 4.6. Cell Seeding on 2D WPI Scaffolds

Flat, round samples with different WPI concentrations (ranging from 15 to 40%) were prepared via punching and placed in the 24-well plates. HUVECs were seeded on top of the scaffolds (at 2.5 × 10^4^ cells/scaffold) in endothelial cell medium and incubated in a humidified atmosphere at 95% relative humidity and 7.5% CO_2_ at 37 °C for 1 or 7 days. Culture media were changed 24 h after seeding and then every second day.

### 4.7. Cell Staining and Image Analysis

Cell morphology was investigated on Day 1 and Day 7. After the required incubation period, cells were fixed with 4% buffered paraformaldehyde and permeabilized with 0.2% Triton X-100 in PBS. F-actin was stained with rhodamine phalloidin (Thermo Fisher) at a final concentration of 200 units/mL, and nuclei were visualized using Hoechst 33,342 (ThermoFischer) at a final concentration of 0.5 µM. Images were taken with Zeiss Axio Observer Z1 microscope (Zeiss, Jena, Germany) with 10× and 20× magnification.

### 4.8. Mitochondrial Activity

Mitochondrial activity of cells growing on WPI scaffolds was assessed after 1 and 7 days of cultivation using WST-8 assay. The assay is based on the extracellular reduction of tetrazolium salt WST-8 by NADH dehydrogenases produced in the mitochondria of viable cells to a water-soluble orange formazan, which dissolves directly into the culture medium. Media were completely removed from the wells and endothelialized hydrogel samples were transferred into the new wells with freshly prepared culture medium containing 1 v% WST-8 reagent (Promocell, Heidelberg, Germany). Following incubation for 2 h, 100 µL of supernatant from each sample was transferred into a well of a 96-well plate and the absorbance at 450 nm was measured in triplicate with a microplate reader.

### 4.9. Compressive Testing of Hydrogels

WPI-20% hydrogels were subjected to compressive testing. Briefly, cylindrical samples of height 10 mm and diameter 8 mm were prepared according to the procedure described earlier in this chapter. Compressive testing was performed using a Zwick 1435 Universal Testing Machine (Zwick, Ulm, Germany) with a testing speed of 10 mm/min within a testing range of 0–80% strain. Young’s modulus, compressive strength and compressive strain at break were determined. Six identical samples were tested, and the mean value and standard deviation were calculated.

### 4.10. SEM Analysis of Hydrogels

The shape and appearance of the hydrogels (surface and cross-sections) were analyzed using a scanning electron microscope (SEM; Auriga, Zeiss, Oberkochen, Germany). All samples were freeze dried using Alpha 1–2 LD plus (Martin Christ, Osterode am Harz, Germany) attached to an aluminum specimen holder using a carbon tape. Images were acquired at different magnifications with an acceleration voltage of 1 kV.

### 4.11. Preparation of Tubular Scaffolds

**Figure 8:**
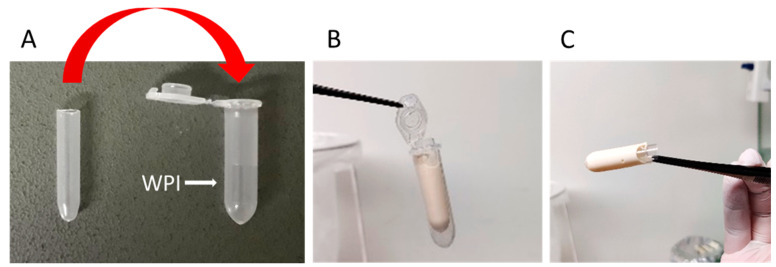
Preparation of WPI-20% tubes. (**A**) Tube-in-tube set-up; (**B**) The hydrogel form after autoclaving; (**C**) The resulting 3D tubular scaffold.

Tubular hydrogels were formed by inserting a tube-like structure into WPI solution inside the Eppendorf tube (Figure 8), which served as a mold. Weighed WPI powder was added to endothelial cell growth media slowly to obtain 20% (*w/v*) solution and stirred overnight at room temperature to dissolve the powder. WPI solution was then cast in the space between the tube walls and autoclaved at 121 °C for 30 min to solidify the tube form. After autoclaving, the Eppendorf tubes containing tubular hydrogels were tightly closed under sterile conditions until further investigation. The resulting tubular scaffolds had a final inner diameter of 7 mm.

### 4.12. Rotational Cell Seeding on 3D Scaffolds

Tubular scaffolds were placed into tissue culture tubes (TPP, Switzerland) and primary HUVECs were introduced to the system at a final density of 10 × 10^5^ cell/mL. Tubes were placed on a roller mixer for 40 min to allow for endothelial cell attachment. Afterwards, tubes were transferred into 37 °C CO_2_ incubator. Cell attachment and morphology were investigated on Days 1, 3 and 7 using F-actin and nuclei staining as described above.

### 4.13. Magnetic Cell Seeding on the Tubular Scaffolds

The produced tubular WPI scaffolds with an inner diameter of 7 mm were placed in the transparent plastic tubes. HUVECs grown in cell culture flasks were pre-incubated with lauric acid-coated SPIONs (3 µg Fe/cm^2^) for 24 h at 37 °C, as described in the previously reported dose-finding studies [45]. After incubation, the SPION-loaded cells were harvested and centrifuged, followed by cell counting. HUVECs (7.5 × 10^5^ cells) were suspended in the culture media and transferred into the luminal space of scaffolds. Immediately after transferring the cells, the scaffolds were exposed to a radial magnetic field for 15 min using a VascuZell endothelizer (Vascuzell Tecnologia S.L., Madrid, Spain [46,47]). The scaffold-containing tubes were then carefully removed from the endothelizer and placed in the incubator for 24 h or 7 days of cultivation. The culture medium was changed 24 h after seeding and then every second day.

### 4.14. Statistical Analysis

All experiments were repeated independently three times and included a minimum of triplicate samples. Data are presented as mean ± standard error of mean (SEM). The analysis of differences between the groups was conducted using Kruskal–Wallis One Way Analysis of Variance on Ranks or Mann–Whitney Rank Sum Test, depending on the results of the normality test. *p*-value < 0.05 was considered statistically significant.

## 5. Conclusions

Our findings indicate that for multiple regenerative purposes, WPI hydrogels have a very high potential for vascularization upon in vivo implantation due to their good biocompatibility with human endothelial cells. Because endothelial monolayer formation was comparable over the range of WPI concentrations, the tuning of the hydrogel properties to the osteoblastic cells for the purposes of, e.g., bone regeneration, should not prevent subsequent vascularization over time. Further studies concerning angiogenic responses in the presence of bone-forming cells will be necessary to evaluate the possibility of WPI scaffold vascularization in more physiologic-like conditions.

## Data Availability

Data sets generated during the study will be available upon request.
